# Hypoxic and pharmacological activation of HIF inhibits SARS-CoV-2 infection of lung epithelial cells

**DOI:** 10.1016/j.celrep.2021.109020

**Published:** 2021-04-05

**Authors:** Peter A.C. Wing, Thomas P. Keeley, Xiaodong Zhuang, Jeffrey Y. Lee, Maria Prange-Barczynska, Senko Tsukuda, Sophie B. Morgan, Adam C. Harding, Isobel L.A. Argles, Samvid Kurlekar, Marko Noerenberg, Craig P. Thompson, Kuan-Ying A. Huang, Peter Balfe, Koichi Watashi, Alfredo Castello, Timothy S.C. Hinks, William James, Peter J. Ratcliffe, Ilan Davis, Emma J. Hodson, Tammie Bishop, Jane A. McKeating

**Affiliations:** 1 Nuffield Department of Medicine, University of Oxford, Oxford, UK; 2 Chinese Academy of Medical Sciences (CAMS) Oxford Institute (COI), University of Oxford, Oxford, UK; 3 Ludwig Institute for Cancer Research, University of Oxford, Oxford, UK; 4 Department of Biochemistry, University of Oxford, Oxford, UK; 5 Respiratory Medicine Unit and National Institute for Health Research (NIHR) Oxford Biomedical Research Centre (BRC), Nuffield Department of Medicine, Experimental Medicine, University of Oxford, Oxford, UK; 6 Sir William Dunn School of Pathology, University of Oxford, Oxford OX1 3RE, UK; 7 MRC-University of Glasgow Centre for Virus Research, Glasgow, UK; 8 Peter Medawar Building for Pathogen Research, Department of Zoology, University of Oxford, Oxford, UK; 9 Research Center for Emerging Viral Infections, College of Medicine, Chang Gung University, Taoyuan, Taiwan; 10 Division of Pediatric Infectious Diseases, Department of Pediatrics, Chang Gung Memorial Hospital, Taoyuan, Taiwan; 11 Department of Virology II, National Institute of Infectious Diseases, Tokyo 162-8640, Japan; 12 Department of Applied Biological Science, Tokyo University of Science, Noda 278-8510, Japan; 13 Francis Crick Institute, London, UK; 14 Department of Experimental Medicine and Immunotherapeutics, University of Cambridge, Cambridge, UK

**Keywords:** hypoxia, HIF, SARS-CoV-2, HIF prolyl hydroxylase inhibitor

## Abstract

COVID-19, caused by the novel coronavirus SARS-CoV-2, is a global health issue with more than 2 million fatalities to date. Viral replication is shaped by the cellular microenvironment, and one important factor to consider is oxygen tension, in which hypoxia inducible factor (HIF) regulates transcriptional responses to hypoxia. SARS-CoV-2 primarily infects cells of the respiratory tract, entering via its spike glycoprotein binding to angiotensin-converting enzyme 2 (ACE2). We demonstrate that hypoxia and the HIF prolyl hydroxylase inhibitor Roxadustat reduce ACE2 expression and inhibit SARS-CoV-2 entry and replication in lung epithelial cells via an HIF-1α-dependent pathway. Hypoxia and Roxadustat inhibit SARS-CoV-2 RNA replication, showing that post-entry steps in the viral life cycle are oxygen sensitive. This study highlights the importance of HIF signaling in regulating multiple aspects of SARS-CoV-2 infection and raises the potential use of HIF prolyl hydroxylase inhibitors in the prevention or treatment of COVID-19.

## Highlights

• Hypoxia downregulates SARS-CoV-2 receptors ACE2 and TMPRSS2 and inhibits viral entry
• Hypoxic signaling inhibits SARS-CoV-2 replication and particle genesis via HIF-1α
• HIF prolyl hydroxylase inhibitors are a potential therapeutic option for COVID-19

## Graphical abstract

**Figure:**
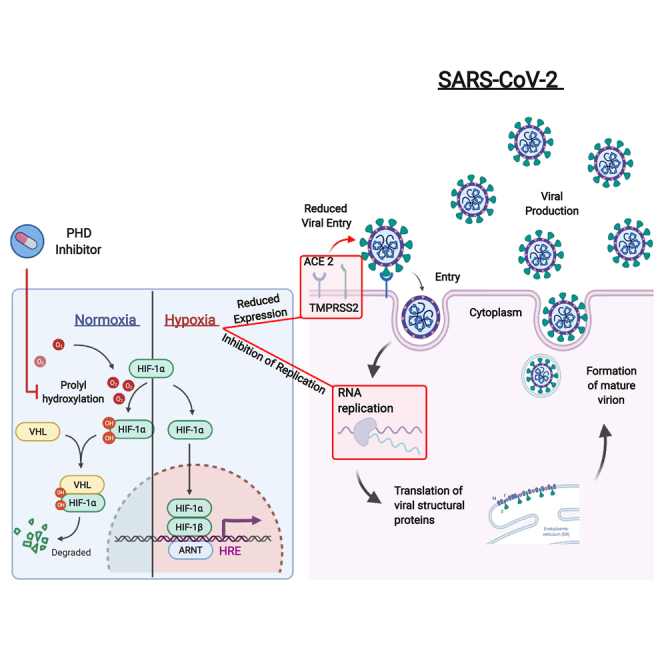

## Teaser

Wing et al. demonstrate that key aspects of the SARS-CoV-2 life cycle are dependent on cellular oxygen tension. Activation of the cellular oxygen-sensing pathway inhibits SARS-CoV-2 infection, highlighting a key cellular pathway that could be exploited as a potential therapeutic avenue for COVID-19.

## Main text

The COVID-19 pandemic, caused by the novel coronavirus SARS-CoV-2, is a global health issue. Although multiple public health approaches, including mass vaccination and social distancing, are needed to bring the pandemic under control, there is an urgent need for prophylactic measures or early treatment that can be targeted to vulnerable groups. This is of particular importance given the emergence of SARS-CoV-2 variants and the uncertainty of future vaccine efficacy. SARS-CoV-2 primarily targets the respiratory tract and infection is mediated by spike (S) protein binding to the human angiotensin-converting enzyme 2 (ACE2), where the transmembrane protease serine 2 (TMPRSS2) triggers fusion of the viral and cell membranes (Hoffmann et al., 2020; Wan et al., 2020). ACE2 is highly expressed in epithelial cells of the respiratory tract as well as those of the kidney and intestine (Hamming et al., 2004; Tipnis et al., 2000; Zhao et al., 2020b). Although COVID-19 is mild in most cases, a defining feature of severe disease is systemic low-oxygen levels (hypoxemia), which is often disproportionate to lung injury. There is evidence to suggest that this profound hypoxemia may alter the ability of SARS-CoV-2 to infect host cells. Hypoxia has been reported to regulate the replication of a number of viruses (Jiang et al., 2006; Kraus et al., 2017; Zhao et al., 2020a; Zhuang et al., 2020), enhancing the replication of Epstein-Barr virus (Jiang et al., 2006; Kraus et al., 2017), but suppressing HIV and influenza infection (Zhao et al., 2020a; Zhuang et al., 2020), demonstrating that the interaction between hypoxia signaling and viral infection is context specific and dependent on both the host cell and viral species. Furthermore, hypoxia has been reported to either induce or, in some cases, suppress ACE2 expression in lung pulmonary arterial smooth muscle cells (PASMCs) (Zhang et al., 2009, 2019), hematopoietic stem cell precursors (Joshi et al., 2019), and hepatocarcinoma cells (Clarke et al., 2014). Because the effects of low oxygen on both ACE2 expression and SARS-CoV-2 replication are likely to be cell context dependent, we evaluated whether hypoxia alters SARS-CoV-2 entry and replication in lung epithelial cells.

Mammalian cells adapt to low oxygen through an orchestrated transcriptional response regulated by hypoxia-inducible factor (HIF), a heterodimeric transcription factor comprising HIF-1α or HIF-2α subunits, which is regulated by oxygen-dependent and -independent stress signals. When oxygen is abundant, newly synthesized HIFα subunits are rapidly hydroxylated by HIF prolyl-hydroxylase domain (PHD) enzymes and are targeted for polyubiquitination and proteasomal degradation. In contrast, when oxygen is limited, HIFα subunits translocate to the nucleus, dimerize with HIF-1β, and activate the transcription of genes involved in cell metabolism, proliferation, pulmonary vasomotor control, and immune regulation (Kaelin and Ratcliffe, 2008; Palazon et al., 2014; Urrutia and Aragones, 2018). Defining how hypoxia or activation of HIF affects the SARS-CoV-2 life cycle in lung epithelial cells will increase our understanding of disease pathogenesis and inform therapeutic strategies. Specifically, this has the potential for pharmacological intervention because drugs that inhibit the PHD enzymes to stabilize HIF (Pugh and Ratcliffe, 2017; Sanghani and Haase, 2019) are either in advanced clinical trials for the treatment of renal anemia or are licensed for clinical use (Roxadustat in China [Chen et al., 2019a, 2019b) and Japan [Akizawa et al., 2020a, 2020c, 2020d] and Daprodustat in Japan [Akizawa et al., 2020b]).

**Figure 1:**
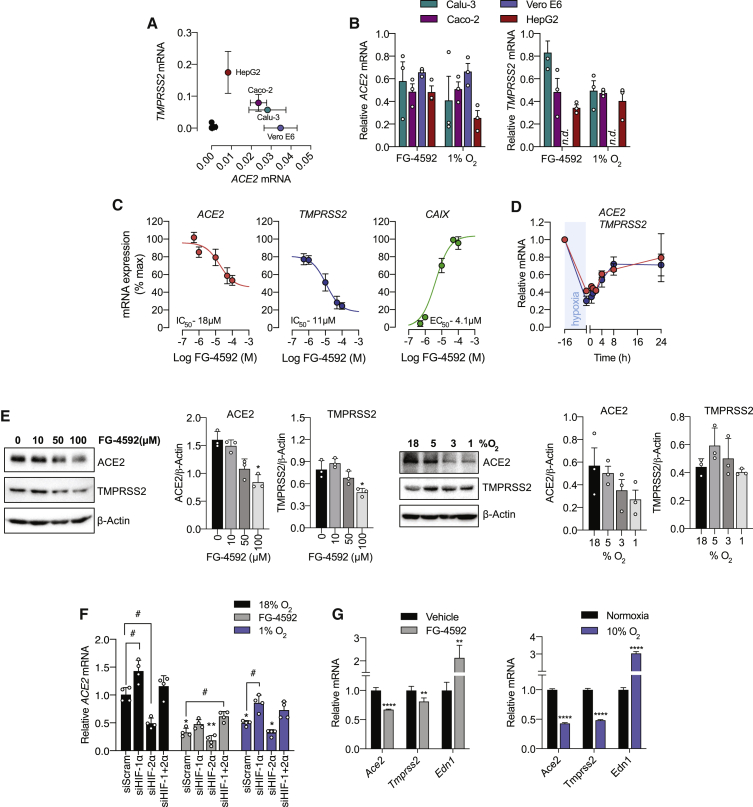
Hypoxia or FG-4592 (Roxadustat) inhibits the expression of SARS-CoV-2 entry factors *in vitro* and *in vivo* (A) *ACE2* and *TMPRSS2* transcript levels across a panel of cell lines: HepG2 hepatoma, SH-SY5Y neuronal, RKO colon epithelial, Caco-2 colon epithelial, U937 monocyte/macrophage, Vero E6 monkey epithelial kidney, Calu-3 airway epithelial, A549 airway epithelial, EA.hy926 umbilical vein endothelial, and U-2OS osteosarcoma endothelial. Cells with minimal *ACE2* and *TMPRSS2* mRNA expression (SH-SY5Y, RKO, U937, A549, EA.hy926, and U-2OS) are displayed as black dots. Data are expressed relative to *HPRT* (hypoxanthine-guanine phosphoribosyl transferase). (B) *ACE2-*expressing cell lines from (A) were treated with FG-4592 (50 μM) or 1% O_2_ for 24 h, and *ACE2* and *TMPRSS2* mRNA was assessed. Data are presented relative to untreated cells; n.d., not detected. (C) HepG2 cells were treated with increasing concentrations of FG-4592 for 24 h, and *ACE2*/*TMPRSS2* mRNA was quantified and expressed as a percentage of the maximal induction/inhibition. *CAIX* mRNA levels were analyzed in parallel as an established HIF-1α-regulated host gene. (D) HepG2 cells were cultured at 1% O_2_ for 16 h and were re-oxygenated over a 0.5–24-h period, and *ACE2*/*TMPRSS2* mRNA levels were analyzed at the indicated times. (E) Calu-3 cells were treated with an increasing concentration of FG-4592 (0–100 μM) or 18%, 5%, 3%, and 1% O_2_ for 24 h, and ACE2/TMPRSS2 protein expression was assessed by immunoblot. Densitometric values are expressed relative to β-actin. (F) siRNAs targeting either HIF-1 or 2α were delivered into Calu-3 cells individually or in combination. Cells were treated with FG-4592 (50 μM) or 1% O_2_ for 24 h, and *ACE2* mRNA levels were quantified. Data are expressed relative to the normoxic siScramble (siScram) control. Statistical significance was determined by two-way ANOVA. ^∗^denotes significance relative to normoxic siRNA (siScram) at 18% O_2,_ whereas indicates significance relative to the control siRNA per condition. (G) C57BL/6 mice were treated with FG-4592 (oral, 10 mg/kg twice daily) or 10% O_2_ for 24 h, and mRNA expression of *Ace2*, *Tmprss2*, and HIF target gene *Edn1* in the lungs was determined by qPCR. mRNA expression for each target gene (relative to *ActB*) was compared between biological groups using a Student’s two-tailed t test, ^∗∗^p < 0.01, ^∗∗∗∗^p < 0.0001. Data are presented as means ± SD from (A–D), n = 3–6; (E) n = 3; (F) n = 4; and (G) n = 4 (n = 3 for *Ace2* in FG-4592 or vehicle-treated mice). See also Figure S1.

The host proteins ACE2 and TMPRSS2 are key determinants of SARS-CoV-2 cell entry (Hoffmann et al., 2020). We screened several commonly used cell lines for *ACE2* and *TMPRSS2* mRNA, and only four demonstrated notable expression of *ACE2*: HepG2 (hepatoma), Caco-2 (colonic adenocarcinoma), Calu-3 (lung adenocarcinoma), and Vero E6 (monkey kidney epithelia) (Figure 1A). We noted that Vero E6 do not express TMPRSS2 mRNA. To assess the role of HIF in regulating these entry factors, we cultured the cells under hypoxic conditions (1% O_2_) or after being treated with an inhibitor targeting the PHD enzymes (FG-4592/Roxadustat), which stabilizes HIFα subunits and upregulates HIF target gene transcription. Both treatments reduced *ACE2* and *TMPRSS2* transcripts, with the magnitude of effect varying between cell lines (Figure 1B). Successful activation of the HIF-signaling pathway was confirmed by induction of the HIF target genes carbonic anhydrase IX (*CAIX*), N-Myc downstream regulated 1 (*NDRG1*), and Egl-9 homolog or HIF prolyl hydroxylase 3 (*EGLN3* or *PHD3*) (Figure S1A). In HepG2 cells, in which transcript suppression was most evident, FG-4592 downregulated *ACE2* and *TMPRSS2* mRNA levels in a dose-dependent manner concomitant with its induction of *CAIX*, *NDRG1*, and *EGLN3* transcription (Figures 1C and S1B). Reoxygenation of cells previously exposed to hypoxia led to a recovery of both *ACE2* and *TMPRSS2* mRNA to near pre-hypoxic levels (Figure 1D), suggesting a specific action of the HIF-PHD pathway. To assess whether hypoxia/FG-4592 regulation is evident at the protein level, we also measured ACE2 and TMPRSS2 protein expression in human lung epithelial Calu-3 cells, a more physiologically relevant cell type for studying SARS-CoV-2 infection. Culturing Calu-3 cells under hypoxic conditions or treating with FG-4592 significantly reduced ACE2 protein expression in a dose-dependent manner with maximum suppression >50 μM FG-4592 or <3% oxygen (Figure 1E) and no effect on cell viability (Figure S1C). Similar, but more modest, effects were observed with TMPRSS2 expression (Figure 1E). The hypoxia-induced changes in ACE2 (and, to a lesser extent, TMPRSS2) protein expression were observed in HepG2 cells (Figure S1D). Any differences between mRNA and protein levels may, in part, reflect the cleavage and secretion of the TMPRSS2 catalytic domain or that additional hypoxia-stimulated factors regulate protein stability and/or expression. To assess the role of HIF, we silenced HIF-1α or HIF-2α expression in hypoxic or FG-4592-treated Calu-3 cells with small interfering RNAs (siRNAs). siRNA-mediated silencing of HIF-1α (either alone or in combination with HIF-2α) restored *ACE2* mRNA levels in FG-4592-treated or hypoxic Calu-3 cells (Figure 1F). In contrast, silencing HIF-2α did not restore *ACE2* mRNA levels in either condition tested and resulted in a modest decrease under normoxic conditions (Figure 1F). siRNA knockdown was verified by quantifying the relevant HIFα transcripts *CAIX*, *NDRG1*, *EGLN3*, and *VEGFA* (Figure S2). These data reveal a role for HIF-1α in repressing ACE2 mRNA and protein expression.

To expand these observations to an *in vivo* setting, mice were treated with hypoxia (10% O_2_) or FG-4592 for 24 h, with a dosing regimen (oral, 10 mg/kg twice daily) similar to that previously used to induce polycythemia (Schley et al., 2019) and the clinical dose for treating renal anemia (Provenzano et al., 2016). Both treatments reduced *Ace2* and *Tmprss2* transcripts in the lung, along with an increase in Endothelin 1 (*Edn1*) mRNA (Figure 1G), a host gene previously reported to be induced by HIF activation in the respiratory tract (Hickey et al., 2010). Collectively, these data show a role for hypoxia in reducing *ACE2* and *TMPRSS2 in vitro* across multiple cell lines, and this is recapitulated in the lungs of mice after systemic hypoxia or FG-4592 treatment.

**Figure 2:**
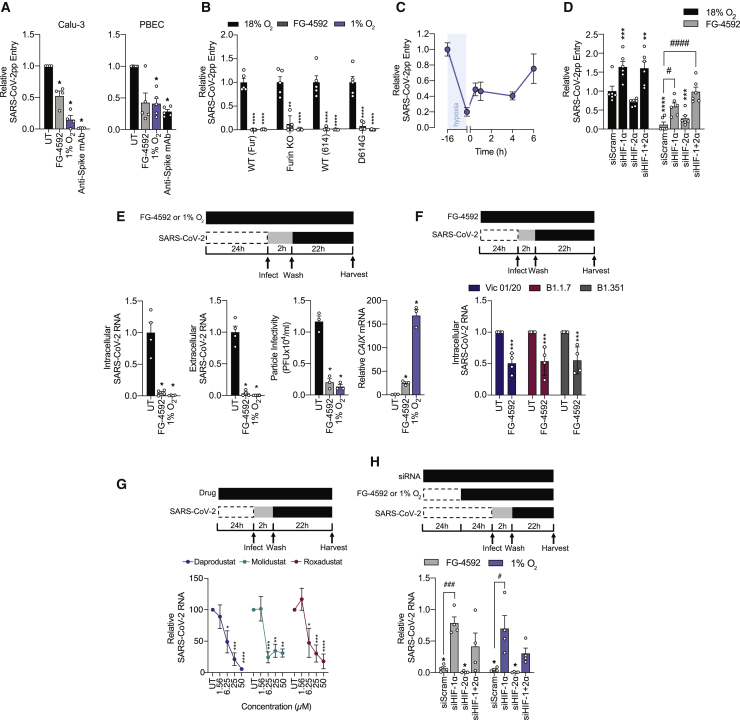
Hypoxia or FG-4592 (Roxadustat) inhibits SARS-CoV-2 entry in a HIF-1α-dependent manner (A) Calu-3 and primary bronchial epithelial cells (PBECs) pre-treated for 24 h with either FG-4592 (50 μM) or 1% O_2_ were infected with SARS-CoV-2 pseudoparticle (pp), and infection was measured after 48 h. To demonstrate the specificity of entry via the spike protein, the pp was incubated with anti-spike monoclonal antibody (mAb) FI-3A (1 μg/mL) for 30 min before infection. Data are expressed relative to untreated (UT) cells. (B) Calu-3 cells were treated with FG-4592 (50 μM) or 1% O_2_ and infected with wild-type (WT) or mutant (D614G or Furin knockout [KO]) SARS-CoV-2pp, and infection was measured 48 h later. Data are expressed relative to UT cells. (C) Calu-3 cells were cultured at 1% O_2_ for 16 h and re-oxygenated over a 0.5–6-h period. Cells were infected with SARS-CoV-2pp at the indicated times, and the pp entry levels were measured 48 h after infection. Data are expressed relative to normoxic cells. (D) siRNAs against HIF-1α and HIF-2α were delivered into Calu-3 cells either individually or in combination. Cells were treated with FG-4592 (50 μM) 24 h after transfection and then infected with SARS-CoV-2pp. Data are expressed relative to an siScrambled (siScram) control. ^∗^ denotes significance relative to control siRNA (siScram) at 18% O_2_, whereas # indicates significance relative to control siRNA per condition. (E) Calu-3 cells were treated with FG-4592 (50 μM) or cultured at 1% O_2_ for 24 h before inoculation with SARS-CoV-2 (MOI 0.001) for 2 h. Infected cells were washed to remove the residual inoculum, and viral replication was assessed 24 h after infection by measuring intracellular and extracellular viral RNA along with infectious titer (particle infectivity) through quantification of plaque-forming units (PFU)/mL. As a control to measure the cellular response to FG-4592 or 1% O_2_, *CAIX* mRNA was quantified by qPCR. All data (except particle infectivity) is expressed relative to the UT control. (F) Calu-3 cells were treated with FG-4592 (50 μM) for 24 h before inoculation with SARS-CoV-2 Victoria 01/20, B1.1.7, or B1.351 (MOI 0.003) for 2 h. Infected cells were washed to remove the residual inoculum, and viral replication was assessed 24 h after infection by measuring intracellular viral RNA, and data are expressed relative to the UT control. (G) Calu-3 cells were treated with increasing concentrations of Daprodustat (GSK: 1278863), Molidustat (Bay 85-3934), and Roxadustat (FG-4592) and were infected with SARS-CoV-2, and viral replication was assessed 24 h later. Data are expressed relative to UT cells. (H) siRNA targeting either HIF-1 or 2α was delivered into Calu-3 cells individually or in combination, and 24 h after transfection was treated with FG-4592 (50 μM) or 1% O_2_ before inoculating with SARS-CoV-2 (MOI 0.001). Intracellular RNA was quantified 24 h after infection, and data are expressed relative to the normoxic siScramble (siScram) control. ^∗^ denotes significance relative to the control siRNA (siScram) at 18% O_2_, whereas # indicates significance relative to control siRNA per condition. Data are presented as means ± SD from (A) n = 4 (Calu-3) and n = 5 (PBEC donors); and (B–G) n = 4. Statistical significance was determined using a one-way (A, B, E, and G) or two-way (D and F) ANOVA. ^∗^p or #p < 0.05, ^∗∗^p or ##p < 0.01, ^∗∗∗^p or ###p < 0.001, ^∗∗∗∗^p or ####p < 0.0001. See also Figures S2–S4.

We hypothesized that the HIF-dependent reduction in ACE2 expression would limit SARS-CoV-2 entry into naive target cells. To assess that, we used lentiviral pseudoparticles (pp) expressing SARS-CoV-2-encoded spike glycoprotein and confirmed that infectivity was ACE2 dependent by infection of human embryonic kidney cells engineered to express ACE2 (Figure S3A). Culturing Calu-3 or primary bronchial epithelial cells (PBECs) under hypoxic conditions or treating with FG-4592 significantly reduced SARS-CoV-2pp infection (Figure 2A). In contrast, viral pp expressing the vesicular stomatitis virus glycoprotein (VSV-G) infected Calu-3 cells and PBEC with comparable efficiency at both oxygen levels (Figure S3B), demonstrating a SARS-CoV-2-specific phenotype. We next sought to test whether hypoxia/FG-4592 limits entry of the novel SARS-CoV-2 spike protein variants; these have emerged throughout the course of the pandemic, with some conferring a fitness advantage to viral entry. The most notable of these to date, D614G, is globally prevalent in the pandemic, consistent with a reported fitness advantage for infecting cells in the upper respiratory tract (Weissman et al., 2021; Korber et al., 2020). Further, deletion of the unique furin cleavage site (which mediates membrane fusion) in the SARS-CoV-2 spike protein has been observed *in vitro* (Davidson et al., 2020) and in animal models of infection (Peacock et al., 2020). Importantly, hypoxia or FG-4592 treatment of Calu-3 cells reduced infection of pp containing either the spike variant to a similar degree as the wild type (Figure 2B). Reoxygenation of hypoxic Calu-3 cells induced a recovery of SARS-CoV-2pp entry (Figure 2C), consistent with our earlier data showing post-hypoxic recovery of *ACE2* and *TMPRSS2* mRNA levels. Silencing HIF-1α reversed the anti-viral effect of FG-4592 (Figure 2D), demonstrating that HIF-1α represses SARS-CoV-2 entry, consistent with its role in regulating *ACE2*. In contrast, we observed a negligible effect of silencing HIF-2α on SARS-CoV-2 entry (Figure 2D). In summary, these data show that hypoxic/FG-4592 activation of HIF-1α represses ACE2 and impairs entry of SARS-CoV-2 entry pp.

We next assessed whether our observations with SARS-CoV-2pp translate to authentic viral replication. Infecting hypoxic (1% O_2_) Calu-3 cells with SARS-CoV-2 (Victoria 01/20 strain) resulted in a 90% reduction in viral RNA compared with that of normoxic cells (Figure 2E). A similar repression in SARS-CoV-2 RNA levels was also observed when culturing Calu-3 cells in 3% oxygen (Figure S4A). Importantly, FG-4592 (50 μM) mimicked the hypoxic inhibition of SARS-CoV-2 replication, leading to a significant reduction in the genesis of new particles (Figure 2E). To define whether hypoxia altered the infectivity of SARS-CoV-2 particles, we assessed the ratio of RNA copies per plaque-forming unit (PFU), finding no significant difference between virus produced from cells at either 18% O_2_ or 1% O_2_ (9.3 × 10^3^ ± 6.7 × 10^3^ and 2.6 × 10^3^ ± 1.6 × 10^3^ means ± SD. RNA copies/PFU, respectively). Notably, we demonstrated comparable antiviral efficacy of FG-4592 treatment against the recently identified B.1.1.7 (United Kingdom) and B1.351 (South Africa) SARS-CoV-2 variants (Figure 2F). Treating Calu-3 cells with FG-4592 or two additional PHD inhibitors of the same class: Daprodustat and Molidustat, inhibited SARS-CoV-2 replication in a dose-dependent manner with maximal inhibition noted at approximately 6 μM (Figure 2G), which is in the range of reported plasma levels in human subjects after oral administration of these drugs at clinical doses (Provenzano et al., 2016). Efficacy of either PHI treatment or hypoxic culture in the activation of HIF was validated by assessing the induction of *CAIX* mRNA (Figures 2E and S4B). siRNA silencing of HIF-1α, but not HIF-2α, in Calu-3 cells reversed the hypoxic or FG-4592-mediated suppression of viral infection, demonstrating a role for HIF-1α in repressing SARS-CoV-2 RNA replication (Figure 2H). These data show a key role for HIF-1α in repressing ACE2-dependent, authentic SARS-CoV-2 entry and infection.

**Figure 3:**
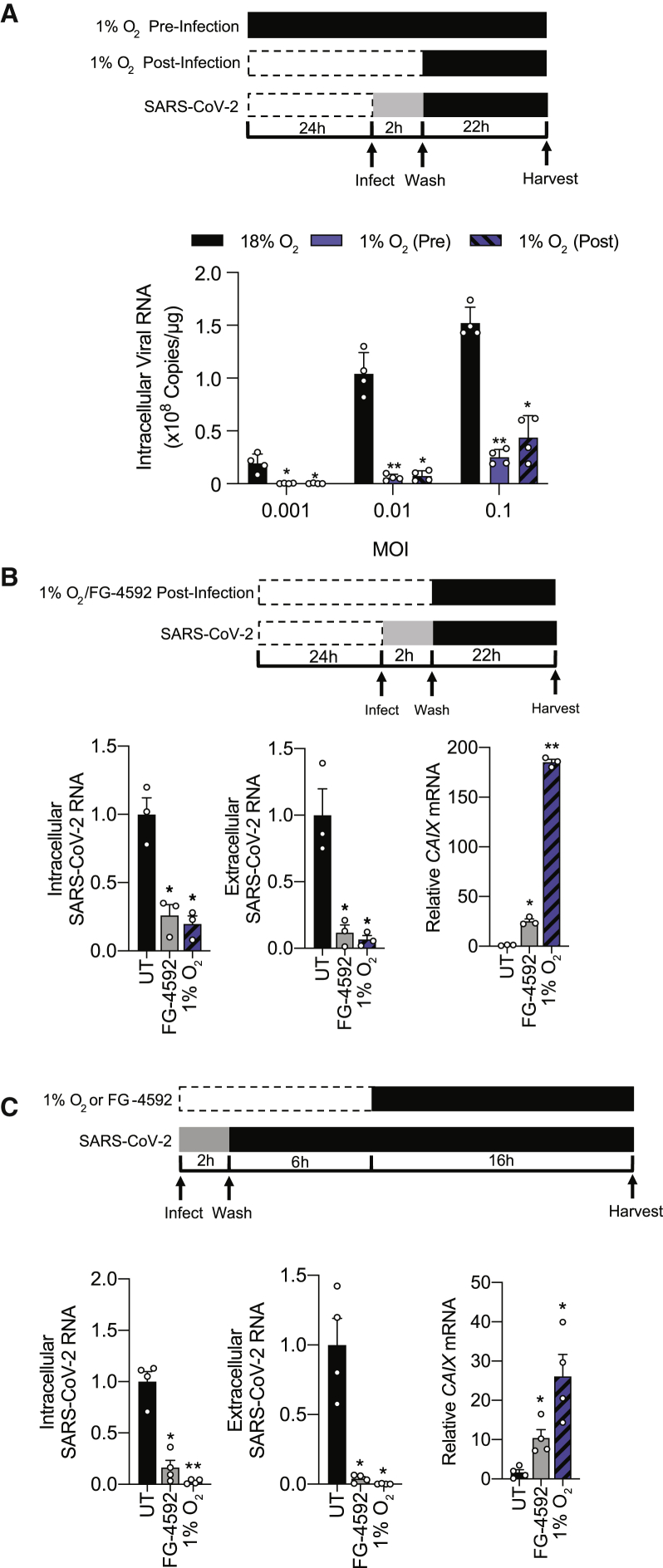
Hypoxia or FG-4592 (Roxadustat) inhibits SARS-CoV-2 replication post-entry (A) Calu-3 cells were treated with 1% O_2_ before or after infection with SARS-CoV-2 at the indicated MOIs, and intracellular RNA was quantified by qPCR 24 h later. Data are expressed as RNA copies × 10^8^/μg of total cellular RNA. (B) Calu-3 cells were inoculated with SARS-CoV-2 (MOI 0.001) for 2 h; unbound virus was removed by washing, and cells were treated with FG-4592 (50 μM) or cultured at 1% O_2_. Viral replication was assessed by measuring intra- and extracellular levels of SARS-CoV-2 RNA. The cellular response to FG-4592 or 1% O_2_ was assessed through *CAIX* mRNA quantification. All data are expressed relative to the UT control. Data are presented as means ± SD from (A and B) n = 4, and statistical significance was determined using a two-way ANOVA. (C) Calu-3 cells were infected with SARS-CoV-2 as detailed above and 8 h later were cultured under 1%O_2_ or treated with FG-4592 (50 μM) for 24 h. Intracellular and extracellular viral RNA, along with *CAIX* transcripts, were measured by qPCR, and data are expressed relative to UT control. Data are presented as means ± SD from (A–C) n = 4, and statistical significance was determined using a two-way ANOVA. ^∗^p < 0.05, ^∗∗^p < 0.01.

To define whether hypoxia signaling regulates additional post-entry steps in the SARS-CoV-2 life cycle, we evaluated the effect of hypoxia on viral replication when applied throughout or after virus inoculation. Hypoxia reduced viral RNA levels in both conditions and at all multiplicities of infection (MOIs) tested (Figure 3A). Importantly, treating SARS-CoV-2-infected Calu-3 with FG-4592 or hypoxia for 24 h significantly reduced both intracellular and extracellular SARS-CoV-2 RNA (Figure 3B). To further define the post-entry effects of HIFs on viral replication, we infected Calu-3 cells and treated them with either FG-4592 or 1% oxygen 8 h later, once replication complexes were established. We noted a significant reduction in intracellular and extracellular viral RNA with both treatments and an induction of *CAIX* mRNA (Figure 3C), demonstrating a role for HIFs in the regulation of post-entry viral RNA replication.

**Figure 4:**
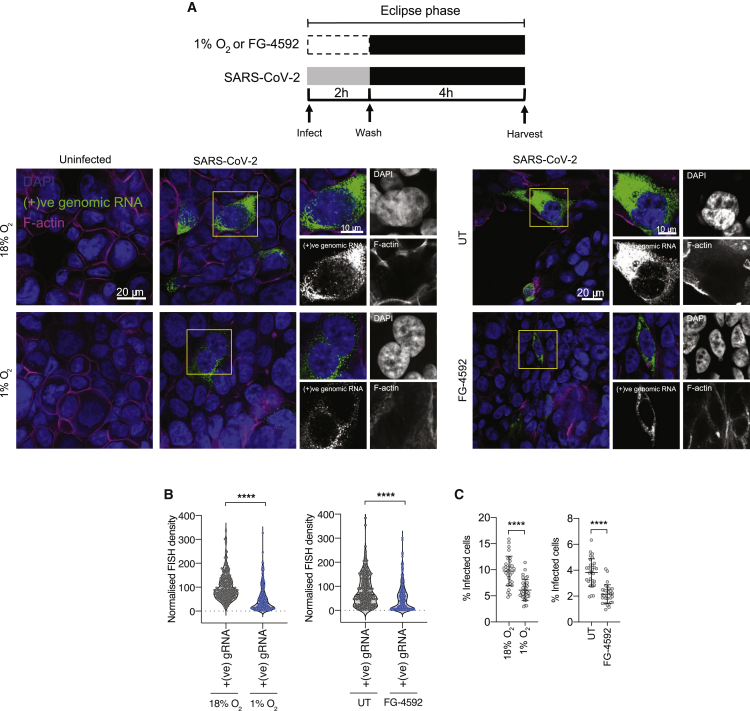
Hypoxia inhibits SARS-CoV-2 RNA replication (A) Calu-3 cells were inoculated with SARS-CoV-2 at an MOI of 1.0 for 2 h; unbound virus was removed by washing, and the cells were cultured at 18% or 1% O_2_ or treated with FG-4592 (50 μM) for 4 h. Cells were fixed, and viral infection was visualized by smFISH, where representative two-dimensional (2D) images depicting positive-strand SARS-CoV-2 genomic RNA are shown. Cells are counter-stained with DAPI to visualize the nucleus; inset images show the individual and merged images, and the scale bar depicts 20 μm. (B) Viral RNA was quantified by integrating the three-dimensional (3D) signal density of individual cells, in which each symbol represents a single cell at 18% or 1% O_2_ or in FG-4592-treated cells. (C) The frequency of SARS-CoV-2 positive-strand RNA expressing cells at the different oxygen levels and after FG-4592 treatment was quantified per field of view, in which each symbol reflects a single field. Data represent the means ± SD percentage of viral RNA derived from n = 3, and significance was assessed by Mann-Whitney test. See also Figure S5.

Given the marked reduction in the cellular viral RNA burden observed under hypoxic conditions, we sought to understand the effect of hypoxia on the initial establishment of viral replication complexes and quantities of positive genomic-strand viral RNA at the single-cell level. Using single-molecule fluorescence in situ hybridization (smFISH), we measured the effect of hypoxia and FG-4592 on positive-strand viral RNAs within the first 6 h of infection, which represents the first cycle of infection (eclipse phase) before the secretion of infectious particles (Figure S5). Hypoxia and FG-4592 treatment significantly reduced the levels of viral RNA per cell (Figures 4A and 4B). We noted a reduction in the frequency of infected cells, as judged by the detection of genomic RNA (Figure 4C). Because de novo generated viral particles were first detected at 6 h after infection (Figure S5), these RNA signals represent primary infection events.

In conclusion, we describe striking inhibitory effects of hypoxia and FG-4592 (Roxadustat) treatment on SARS-CoV-2 entry (including spike variants), replication, and secretion of infectious particles in lung epithelial cells. These effects were mediated by a HIF-1α-dependent repression of SARS-CoV-2 replication, in concert with the reduced expression of *ACE2* across a range of cell lines and mouse lung tissue. Of note, there are reports of hypoxic induction of *ACE2* gene expression in other cell types, albeit often transient (Clarke et al., 2014; Joshi et al., 2019; Zhang et al., 2009). Although this contrasts with our findings, the discrepancy may reflect the minimal *ACE2* expression detected in many cell lines we examined, whereas in this study, we focused on cell lines that express greater levels of *ACE2* and are relevant to the clinical sites of infection. Alternatively, the reported differences in *ACE2* transcriptional regulation may reflect cell-type-specific metabolic phenotypes that modulate HIF signaling (Codo et al., 2020) or expression of co-regulators that mediate indirect effects of HIF stabilization. For example, a study of hypoxic regulation of *ACE2* in PASMCs suggests an indirect mechanism through HIF-1α induction of *ACE1* and ANG-II/ATR1 signaling (Zhang et al., 2009); however, *ACE1* was not regulated by hypoxia or FG-4592 in Calu-3 cells (Figure S2). Interestingly, recent evidence describes a HIF-1α-dependent induction of the microRNA *LET7b*, which directly targets the *ACE2* coding sequence to suppress its expression in hypoxic PASMCs (Zhang et al., 2019). Although the precise mechanism by which HIF-1α represses *ACE2* mRNA in lung epithelial cells is unclear, the reversible nature of this repression, combined with the presence of a hypoxia responsive element in the *ACE2* promoter (Zhang et al., 2009), may be consistent with direct HIF-mediated repression.

Beyond effects on *ACE2*-mediated viral entry, we observed marked suppression of SARS-CoV-2 RNA and genesis of infectious particles by hypoxia or pseudohypoxia. Notably, treatment with additional prolyl hydroxylase inhibitors Daprodustat and Molidustat exhibited a comparable antiviral capacity, suggesting a class effect that extends beyond Roxadustat. HIF has been shown to regulate the replication of other RNA viruses through effects on host cell metabolism (Farquhar et al., 2017; Frakolaki et al., 2018; Zhao et al., 2020a). For example, HIF was reported to repress hepatitis C virus replication in the liver via activation of the autotaxin-lysophosphatidic acid signaling pathway to regulate virus particle genesis (Farquhar et al., 2017). Moreover, our understanding of how HIF regulates respiratory viruses is exemplified by influenza A virus, whose replication was enhanced in mice, with HIF-1α inactivation restricted to type II alveolar epithelial cells (Zhao et al., 2020a), highlighting a role for HIF-1α in repressing this respiratory pathogen. Our findings contrast to those reported by Codo et al. (2020) who showed that treatment of monocytes with the HIF prolyl hydroxylase inhibitor BAY 85-3934 (Molidustat) increased SARS-CoV-2 RNA levels in an HIF-1α-dependent manner. This may relate to cell-type-specific differences; for example, monocytes have limited permissivity to support SARS-CoV-2 replication, and viral RNA levels were substantially lower than those measured from infected lung epithelial cells. Further work is needed to characterize the HIF-1α-dependent mechanisms of SARS-CoV-2 repression described here, which are likely mediated via HIF-1α regulation of host factors essential for viral RNA replication and/or stability.

Our observations raise clear questions as to how cellular hypoxia translates to humans, both in terms of SARS-CoV-2 susceptibility and clinical progression of COVID-19. There has been some speculation that chronic hypoxia may be protective, with reports of reduced incidence of COVID-19 disease in high-altitude human populations (Pun et al., 2020) (although these observations are complicated by geographic and socioeconomic factors). Some clinical studies suggest that smokers and patients with chronic respiratory diseases (e.g., asthma and COPD) are under-represented co-morbidities in hospitalized patients with COVID-19 (Halpin et al., 2020). However, these conditions are also associated with a higher risk of poor outcomes in established infections (Lippi and Henry, 2020; Sanchez-Ramirez and Mackey, 2020) and, more generally, hypoxemia is a negative prognostic indicator in severe COVID-19 (Berenguer et al., 2020; Petrilli et al., 2020; Yadaw et al., 2020). Although this is seemingly at odds with our findings, clinical hypoxemia is a complex state that reflects multiple pathogenic processes, including vascular inflammation, coagulopathy, and microthrombotic disease (McGonagle et al., 2020; Varga et al., 2020), which may confound any protective effects of hypoxia on SARS-CoV-2 infection.

A key finding from our study is the potential therapeutic application of Roxadustat, and other related HIF prolyl hydroxylase inhibitors, in COVID-19, especially because these act on multiple stages of the viral life cycle (impairing entry and replication) and, as such, may be effective against emerging SARS-CoV-2 variants. These drugs have been developed as erythropoiesis-stimulating agents in patients with anemic and chronic kidney disease and are currently being used in both pre-dialysis and dialysis settings. Thus, it is likely that substantial numbers of patients who are at risk of severe COVID-19 (Williamson et al., 2020; Wu et al., 2020) will be receiving these drugs. Our work highlights the urgent need to monitor these patients for any evidence that PHD inhibitors provide prophylactic and/or therapeutic activity against COVID-19. However, clinical translation of Roxadustat may be complex because HIF has multiple systemic effects that could affect COVID-19 disease progression. Moreover, ACE2 is protective in models of lung injury (Kuba et al., 2005), so it is uncertain whether reducing ACE2 expression would have a net benefit in severe lung disease. Regardless of the potential complexity, the marked effects of Roxadustat in protecting naive cells from SARS-CoV-2 entry and in inhibiting viral replication within infected cells merits further evaluation in animal models and consideration for study in human clinical trials.

## STAR★Methods

### Key resources table

**Table:** 

REAGENT or RESOURCE	SOURCE	IDENTIFIER
**Antibodies**		

Rabbit anti-Ace2	Abcam	Cat:Ab108252; RRID:AB_10864415
Mouse anti-TMPRSS2	Santa Cruz Biotechnology	sc-515727
Anti-β-Actin-HRP Conjugate	Abcam	Cat:Ab49900; RRID: AB_867494
Anti-Spike FI-3A	Kind Gift from Prof Alain Townsend	FI-3A

**Bacterial and virus strains**		

SARS-CoV-2 Victoria 01/20, BVIC01 (Caly et al., 2020)	Public Health England	SARS-CoV-2 Victoria 01/20
SARS-CoV-2 B1.1.7: 20I/501Y.V1.HMPP1 (Tegally et al., 2020)	Public Health England	SARS-CoV-2 B1.1.7
SARS-CoV-2 B1.351: 201/501.V2.HV001 (Cele et al., 2021)	Centre for the AIDS Programme of Research in South Africa	SARS-CoV-2 B1.351

**Chemicals, peptides, and recombinant proteins**		

FG-4592 (Roxadustat)	MedChemExpress	HY-13426
GSK1278863 (Daprodustat)	MedChemExpress	HY-17608
BAY 85-3934 (Molidustat)	MedChemExpress	HY-12654

**Critical commercial assays**		

CytoTox 96® Non-Radioactive Cytotoxicity Assay	Promega	G1780

**Deposited data**		

Source data for all figures	Mendeley Data	https://doi.org/10.17632/yvgx2sgsf6.1

**Experimental models: Cell lines**		

RKO	Kind Gift from Professor Ester Hammond	RKO
U2-OS	Kind Gift from Dr. Sebastian Nijman	U2-OS
Caco-2	The Francis Crick Institute Cell Services	Caco-2
Vero-E6	Kind Gift from Professor William James	Vero-E6
SH-SY5Y	Kind Gift from Professor E. Yvonne Jones	SH-SY5Y
Calu-3	Kind Gift from Professor Nicole Zitzmann	Calu-3
U937	The Francis Crick Institute Cell Services	U937
A549	The Francis Crick Institute Cell Services	A549
HepG2	Kind gift from Prof Stephan Urban	HepG2
EA.hy926	Kind Gift from Professor Giovanni Mann	EA.hy926

**Experimental models: Organisms/strains**		

Mouse: wild-type JAX C57BL/6	Charles River/ in-house breeding at the Functional Genetics Facility of the Wellcome Trust Centre for Human Genetics (University of Oxford)	CR Strain code: 632

**Oligonucleotides**		

ACE2 forward: GGGATCAGAGATCGGAAGAAGAAA	This Paper	N/A
ACE2 reverse: AGGAGGTCTGAACATCATCAGTG	This Paper	N/A
TMPRSS2 forward: AGGTGAAAGCGGGTGTGAGG	This Paper	N/A
TMPRSS2 reverse: ATAGCTGGTGGTGACCCTGAG	This Paper	N/A
CAIX forward: CTTGGAAGAAATCGCTGAGG	This Paper	N/A
CAIX reverse: TGGAAGTAGCGGCTGAAGTC	This Paper	N/A
EGLN3 forward: CACGAAGTGCAGCCCTCTTA	This Paper	N/A
EGLN3 reverse: TTGGCTTCTGCCCTTTCTTCA	This Paper	N/A
NDRG1 forward: TTTGATGTCCAGGAGCAGGA	This Paper	N/A
NDRG1 reverse: ATGCCGATGTCATGGTAGGT	This Paper	N/A
VEGFA forward: TTGCCTTGCTGCTCTACCTCCA	This Paper	N/A
VEGFA reverse: GATGGCAGTAGCTGCGCTGATA	This Paper	N/A
HPRT forward: GACCAGTCAACAGGGGACAT	This Paper	N/A
HPRT reverse: AACACTTCGTGGGGTCCTTTTC	This Paper	N/A
HIF-1a forward: TATGAGCCAGAAGAACTTTTAGGC	This Paper	N/A
HIF-1a reverse: CACCTCTTTTGGCAAGCATCCTG	This Paper	N/A
HIF-2a forward: CTGTGTCTGAGAAGAGTAACTTCC	This Paper	N/A
HIF-2a reverse: TTGCCATAGGCTGAGGACTCCT	This Paper	N/A
B2M forward: CTACACTGAATTCACCCCCACTG	This Paper	N/A
B2M reverse: ACCTCCATGATGCTGCTTACATG	This Paper	N/A
SARS-CoV-2_N forward: CACATTGGCACCCGCAATC,	This Paper	N/A
SARS-CoV-2_N reverse:GAGGAACGAGAAGAGGCTTG	This Paper	N/A
Ace2 FAM	Thermo Fisher	Mm01159006_m1
Tmprss2 FAM	Thermo Fisher	Mm00443677_m1
Edn1 FAM	Thermo Fisher	Mm00438656_m1
ActB VIC	Thermo Fisher	Mm01205647_g1
siRNA HIF-1A CCAUAUAHAHAUACACAAAtt	Ambion	s6539
siRNA Epas1 (HIF-2A) GUAACUUCCUAUUCACCAAtt	Ambion	s4700
Silencer Select Negative Control siRNA	Ambion	4390843

**Recombinant DNA**		

pSARS-Spike	Kind gift from Craig Thompson (University of Oxford)	N/A
p8.91 (GAG-POL)	Kind gift from Craig Thompson (University of Oxford)	N/A
pCSFW	Kind gift from Craig Thompson (University of Oxford)	N/A
pcDNA3.1-hACE2	Kind gift from Craig Thompson (University of Oxford)	N/A
pSpike-D614G	Kind gift from Ariel Isaacs and Naphak Modhiran (University of Queensland)	N/A
pSpike-FurKO	Kind gift from Dalan Bailey (University of Queensland)	N/A

**Software and algorithms**		

GraphPad 8	Prism	https://www.graphpad.com

### Resource availability

#### Lead contact

Further information and requests for resources and reagents should be directed to and will be fulfilled by the lead contact, Jane McKeating (jane.mckeating@ndm.ox.ac.uk).

#### Materials availability

This study did not generate new unique reagents.

#### Data and code availability

The authors declare that all data supporting the findings of this study are available in the article. Original data have been deposited to Mendeley Data: https://doi.org/10.17632/yvgx2sgsf6.1.

### Experimental model and subject details

#### Animals

All animal procedures were carried out in accordance with the Animals (Scientific Procedures) Act 1986 Amendment Regulations 2012. Mice were housed in the Functional Genetics Facility of the Wellcome Trust Centre for Human Genetics (University of Oxford) in individually ventilated cages with food and water provided *ad libitum* and on a 13h light/11h dark cycle. Wild-type male mice on a C57BL/6 genetic background, approximately 8 weeks old and littermate controlled were used for the experiments. Mice were treated over the course of 24h with 3 oral gavages of 10mg/kg FG-4592 prepared as a 2.5mg/mL solution in 5mg/mL methyl cellulose, 0.5% Tween80 vehicle (or vehicle alone). Hypoxic mice were housed in a normobaric altitude chamber held at 10% O_2_ with controlled temperature, humidity and carbon dioxide levels and compared against mice held in normoxia. Animals were sacrificed by an overdose of Isoflurane (Primal Critical Care) and exsanguination, after which lungs were collected and immediately frozen in liquid nitrogen.

#### Cell culture

RKO, U2-OS, Caco-2 and Vero E6 cell lines were cultured in standard DMEM; SH-SY5Y cell line in DMEM/F-12; Calu-3 in Advanced DMEM; U937 in RPMI; and A549 in F-12K; all supplemented with: 10% fetal bovine serum, 2mM L-glutamine, 100 U/mL penicillin and 10 μg/mL streptomycin. EA.hy926 and HepG2 cells were cultured in standard DMEM additionally supplemented with endothelial cell growth supplement or non-essential amino acids, respectively. All cell lines were maintained at 37°C and 5% CO_2_ in a standard culture incubator and exposed to hypoxia using an atmosphere-regulated workstation set to 37°C, 5% CO_2_:1%–5% O_2_:balance N_2_ (Invivo 400, Baker-Ruskinn Technologies). Human PBECs were obtained using flexible fiberoptic bronchoscopy under light sedation with fentanyl and midazolam from healthy control volunteers. Participants provided written informed consent. The study was reviewed by the Oxford Research Ethics Committee B (18/SC/0361). Airway epithelial cells were taken by 2mm diameter cytology brushes from 3rd to 5th order bronchi and cultured in Airway Epithelial Cell medium (PromoCell, Heidelberg, Germany) in submerged culture.

#### Viral strains

SARS-CoV-2 strains: Victoria 01/20 (BVIC01) (Caly et al., 2020) (provided by PHE Porton Down after supply from the Doherty Centre Melbourne, Australia); B1.1.7 (Tegally et al., 2020) (20I/501Y.V1.HMPP1) (provided by PHE Porton Down) and B1.351 (201/501.V2.HV001) (Cele et al., 2021) (Centre for the AIDS Programme of Research in South Africa) were passaged in Vero E6 cells.

### Method details

#### SARS-CoV-2 pseudoparticle genesis and infection

SARS-CoV-2 lentiviral pseudoparticles (pp) were generated by transfecting 293T cells with p8.91 (Gag-pol), pCSFW (luciferase reporter) and a codon optimized expression construct pcDNA3.1-SARS-CoV-2-Spike, as previously described (Thompson et al., 2020). The Furin cleavage site mutant was generated by mutagenesis of a pcDNA3.1 based clone expressing a C-terminally flag-tagged SARS-CoV-2 Spike protein (Wuhan-Hu-1 isolate; MN908947.3). The polybasic cleavage site TNSPRRA in SARS-CoV-2 Spike was replaced with the corresponding SARS-CoV variant sequence SLL. The pNBF SARS-CoV2 FL D614G mutant was a kind gift from Dr. Daniel Watterson and Dr. Naphak Modhiran (University of Queensland, Australia) and Furin KO mutant from Dr Daniel Bailey (Pirbright Institute, UK). Supernatants containing viral pp were harvested at 48 and 72h post-transfection. Viral titers were determined by infecting Calu-3 cells with a serial dilution of virus and 48h later measuring cellular luciferase. As a control for non-specific lentivirus uptake, stocks were generated with no envelope glycoprotein (No Env). This control was included in all pp experiments and the luciferase values obtained subtracted from values acquired with the SARS-CoV-2pp. To define spike-dependent infection, SARS-CoV-2pp were incubated with the anti-S-mAb FI-3A (1μg/mL) (Hauang et al., 2020) for 30min prior to infection.

#### SARS-CoV-2 propagation and infection

Naive Vero E6 cells were infected with SARS-CoV-2 at an MOI of 0.003 and incubated for 48-72h until visible cytopathic effect was observed. At this point, cultures were harvested, clarified by centrifugation to remove residual cell debris and stored at −80°C. Viral titer was determined by plaque assay. Briefly, Vero E6 cells were inoculated with serial dilutions of SARS-CoV-2 viral stocks for 2h followed by addition of a semi-solid overlay consisting of 1.5% carboxymethyl cellulose (SIGMA). Cells were incubated for 72h, visible plaques enumerated by fixing cells using amido black stain and plaque-forming units (PFU) per mL calculated. For infection of Calu-3 cells with SARS-CoV-2, cells were plated 24h before infection with the stated MOI. Cells were inoculated for 2h after which the residual inoculum was removed with three PBS washes. Unless otherwise stated, infected cells were maintained for 24h before harvesting for downstream applications.

#### Immunoblotting

Cell lysates were prepared by washing cells with phosphate buffered saline (PBS), then lysing in Igepal lysis buffer (10mM Tris pH 7.5, 0.25M NaCl, 0.5% Igepal) supplemented with Complete TM protease inhibitor cocktail (Sigma Aldrich) at 4°C for 5min, followed by clarification by centrifugation (3min, 12,000 rpm). Supernatant was mixed with Laemmli sample buffer, separated by SDS-PAGE and proteins transferred to polyvinylidene difluoride membrane (Immobilon-P, Millipore). Membranes were blocked in 5% milk in PBS/0.1% Tween-20, then incubated with anti-ACE2 (Abcam ab108252) or anti-TMPRSS2 (SCBT sc-515727) primary antibodies and appropriate HRP-conjugated secondary antibodies (DAKO). Chemiluminescence substrate (West Dura, 34076, Thermo Fisher Scientific) was used to visualize proteins using a ChemiDoc XRS+ imaging system (BioRad). Anti-β-actin-HRP conjugate (Abcam ab49900) and/or Coomassie brilliant blue staining was then used to verify equal protein loading and densitometric analysis performed using ImageJ software (NIH).

#### RT-qPCR

Cells were washed in PBS then lysed using Tri-reagent (Sigma), and mRNA extracted by phase separation. Equal amounts of cDNA were then synthesized using the High Capacity cDNA Kit (Applied Biosystems) and mRNA expression determined using Fast SYBR master mix using a StepOne thermocycler (Applied Biosystems) using the ΔΔC_t_ method. See Key resources table for primer sequences. Frozen lungs were homogenized in RLT buffer (QIAGEN) using a Standard Micro-Homogenizer (ProScientific) and mRNA was extracted using the RNeasy Mini kit (QIAGEN), according to manufacturer’s instructions. Equal amounts of cDNA were synthesized using the QuantiTect Reverse Transcription Kit (QIAGEN) and mRNA expression was quantified in triplicates in a duplex quantitative real-time PCR using TaqMan Fast Advanced Master Mix and *Ace2* FAM (Mm01159006_m1), *Tmprss2* FAM (Mm00443677_m1), *Edn1* FAM (Mm00438656_m1) and *ActB* VIC (Mm01205647_g1) assays (Thermo Fisher). The reaction was carried out in the StepOnePlus Real-Time PCR System (Applied Biosystems). ΔC_T_ was defined as the difference between the Target gene C_T_ and the *ActB* C_T_. –ΔΔC_T_ values were calculated for each replicate as follows: -(FG-4592 ΔC_T_ – Vehicle ΔC_T_) (Livak and Schmittgen, 2001). Fold change in the target gene mRNA expression in each genotype group was expressed as 2^–ΔΔCT^.

#### FISH quantification of SARS-CoV-2 RNA

SARS-CoV-2 single-molecule fluorescence *in situ* hybridization (smFISH): smFISH was carried out as previously reported (Yang et al., 2017) with minor modifications. Briefly, cells were grown on #0 round glass coverslips in 24 well plate and fixed in 4% paraformaldehyde for 30min at room temperature. Cells were permeabilised in PBS/0.1% Triton X-100 (PBST) for 10min at room temperature followed by washes in PBS and 2x SSC. Cells were pre-hybridized in prewarmed (37°C) wash solution (2x SSC, 10% formamide) twice for 20min each at 37°C. Hybridization was carried out in hybridization solution (2x SSC, 10% formamide, 10% dextran sulfate) containing 500nM FISH probes overnight at 37°C. SARS-CoV-2 positive and negative genomic RNA FISH probes were labeled with ATTO633 and ATTO565 (ATTO-Tec), respectively (See Table S1), according to published protocols (Gaspar et al., 2017). Individual probe sequences are listed in supplemental data. After the overnight hybridization, cells were washed for 20min in pre-warmed wash solution at 37°C followed by counterstaining with DAPI (1μg/mL) and Phalloidin-AlexaFluor 488 conjugate (264nM), diluted in wash solution. Cells were then washed once with wash solution for 20min at 37°C and twice with 2xSSC for 5min each at room temperature. Coverslips were dipped in pure water and mounted on slides using Vectashield HardSet (Vector Labs).

#### Image acquisition and analysis

Mounted cells were imaged on an Olympus SoRa spinning disc confocal with Orca Flash4 CMOS camera using 60x silicone oil objective (1.3 NA, UPLSAPO60XS2) or 100x silicone oil objective (1.35 NA, UPLSAPO100XS). Specimens were imaged in at least ten different locations per condition and replicate. 3D-stacked images were taken with voxel size of 80nm x 80nm x 200nm in x:y:z and images were deconvolved with maximum likelihood algorithm using cellSens (5 iterations, default PSF, Olympus). Background subtraction was performed on all channels using rolling ball subtraction (radius = 100px) in ImageJ (National Institutes of Health). smFISH signal was quantified using intensity-based methods by manually segmenting individual infected cells using phalloidin stain on a maximum projected image and integrating signal intensity across all slices within region of interest. Integrated intensity was divided by cell volume to obtain signal density per volume, which was normalized by subtracting average signal density of uninfected cells. Infection frequency was quantified per field of view for each 3D image. To get total number of cells, DAPI channel was Gaussian filtered (radius = 10px) in ImageJ and nuclei were automatically counted using spot tool in Imaris (diameter = 6μm, Bitplane). Infected cells were counted manually.

#### Materials

All reagents and chemicals were obtained from Sigma-Aldrich (now Merck) unless stated otherwise. Roxadustat, Molidustat and Daprodustat were obtained from either Selleckchem or MedChemExpress. See Key resources table for details

### Quantification and statistical analysis

Data was analyzed using GraphPad Prism version 8.0.2 (GraphPad, San Diego, CA, (USA). P values < 0.05 were considered significant; significance values are indicated as ^∗^p < 0.05; ^∗∗^p < 0.01; ^∗∗∗^p < 0.001; ^∗∗∗∗^p < 0.0001. Please see individual figure legends for further details.

## Supplemental information

Document S1. Figures S1–S8 and Table S1

Document S2. Article plus supplemental information
