# Supplementary material for: Hypoxic and pharmacological activation of HIF inhibits SARS-CoV-2 infection of lung epithelial cells
Source: Cell Rep. 2021 Apr 5;35(3):109020. doi: 10.1016/j.celrep.2021.109020 (PMC8020087; doi:10.1016/j.celrep.2021.109020)
Supplement: Document S1. Figures S1–S8 and Table S1 [file mmc1.pdf]

Cell Reports, Volume *35*

Supplemental information

Hypoxic and pharmacological activation of HIF inhibits SARS-CoV-2 infection of lung epithelial cells

Peter A.C. Wing, Thomas P. Keeley, Xiaodong Zhuang, Jeffrey Y. Lee, Maria Prange-Barczynska, Senko Tsukuda, Sophie B. Morgan, Adam C. Harding, Isobel L.A. Argles, Samvid Kurlekar, Marko Noerenberg, Craig P. Thompson, Kuan-Ying A. Huang, Peter Balfe, Koichi Watashi, Alfredo Castello, Timothy S.C. Hinks, William James, Peter J. Ratcliffe, Ilan Davis, Emma J. Hodson, Tammie Bishop, and Jane A. McKeating

Supplemental Figures

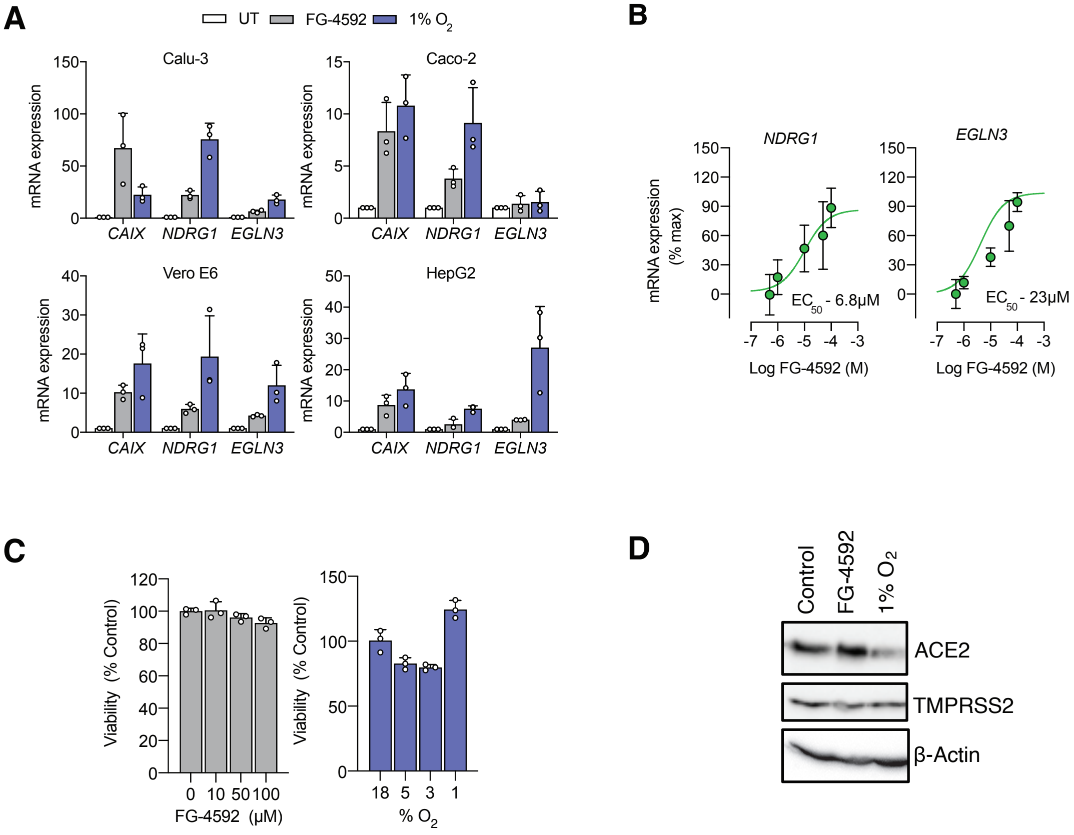

Fig.S1 Hypoxic gene induction in ACE2 expressing cells. [Related to Fig.1] (**A**) Calu-3, Caco-2, Vero E6 and HepG2 cells were treated with FG-4592 (50µM) or 1% O_2 for 24h with *CAIX*, *NDRG1*, and *EGLN3* mRNA assessed by qPCR. Data are expressed relative to normoxic untreated (UT) cells as mean ± S.D. from n=3 biological replicates. (**B**) HepG2 cells were treated with increasing concentrations of FG-4592 for 24h with *NDRG1* and *EGLN3* mRNA quantified. Half-maximal effective concentration (EC_{50}) values for both genes in response to FG-4592 treatment were calculated. (**C**) The impact of either FG-4592 or hypoxic incubation on the viability of Calu-3 cells was assessed through quantification of extracellular lactate dehydrogenase (LDH) 24h post-treatment. **(D)** HepG2 cells were treated with FG-4592 (50µM) or 1% O_2 for 24h and ACE2/TMPRSS2 protein expression assessed by immunoblot. β-Actin was used to show equal protein loading.

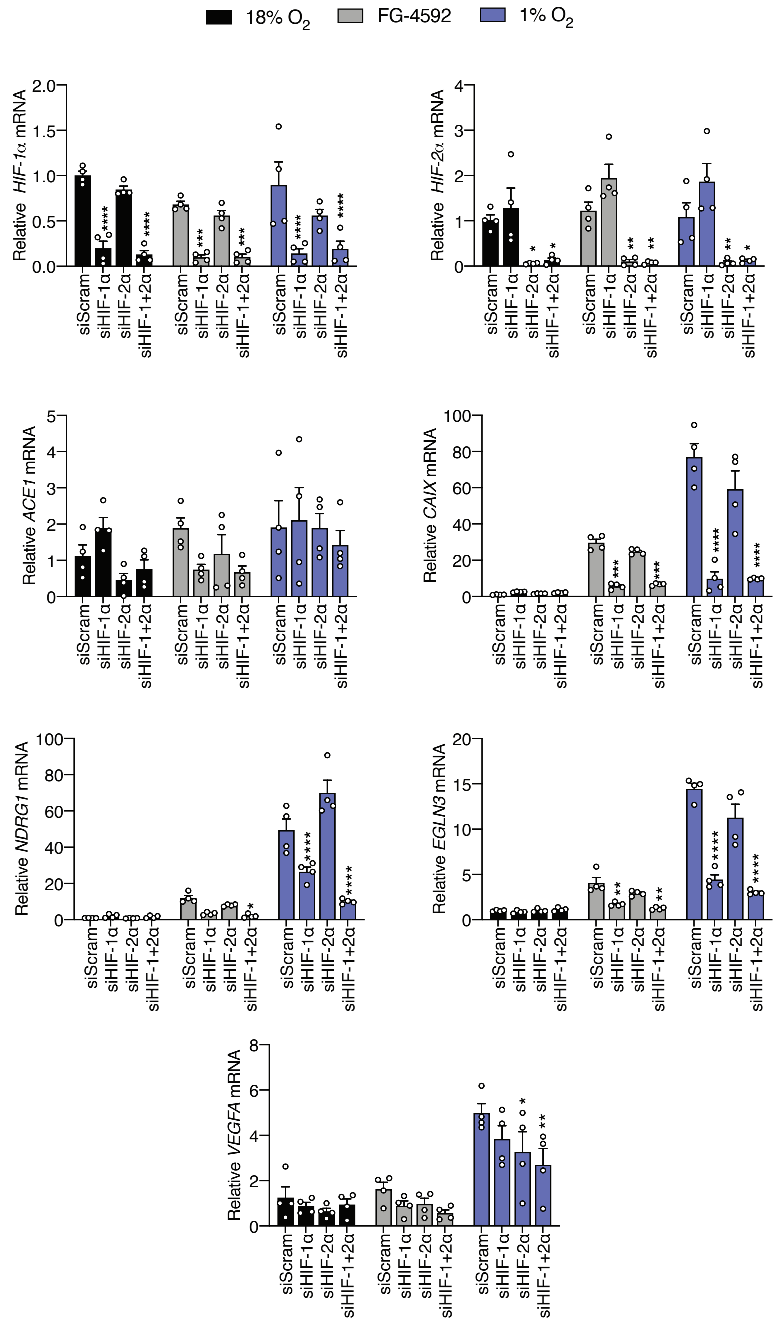

Fig.S2: Validating siRNA silencing of HIF-1α and HIF-2α in Calu-3 cells. [Related to Fig.2].
siRNAs targeting either HIF-1 or 2α were delivered into Calu-3 cells either individually or in combination along with a control scrambled siRNA. 48h post-transfection the cells were treated with FG-4592 (50µM) or 1% O_2 for 24h and total cellular RNA extracted. siRNA knock-down was confirmed by qPCR quantification of *HIF-1α, HIF-2α, ACE1, CAIX, NDRG1, EGLN3* and *VEGFA* mRNA levels. Bars represent mean ± S.D. from n=4 biological replicates and data plotted relative to siScram at 18% O_2 with statistical significance determined by two-way ANOVA, * $p<0.05$ ** $p<0.01$, *** $p<0.001$, **** $p<0.0001$.

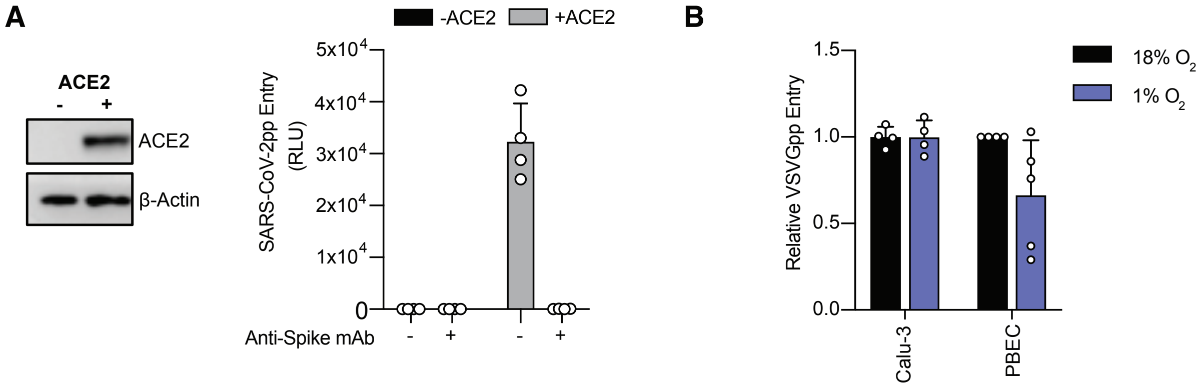

Fig.S3: SARS-CoV-2pp entry is ACE2 dependent. [Related to Fig.2]. (A) Human embryonic kidney 293T cells were transfected with a control or human ACE2 overexpression plasmid and infected with SARS-CoV-2 pseudoparticles (pp) 48h post-transfection. ACE2 expression was confirmed by immunoblot. SARS-CoV-2pp were pre-treated with or without an anti-Spike mAb FI-3A (1µg/ml) for 30min prior to infection. Data is mean ± S.D. from n=4 biological replicates. **(B)** Calu-3 or PBECs were cultured under 18% or 1% O_2 for 24h before infection with viral pseudoparticles expressing vesicular stomatitis virus glycoprotein (VSV-G). Infection was assessed 48h later by quantification of luciferase activity. Data is expressed relative to the normoxic samples and is the mean ± S.D. of n=4 biological replicates.

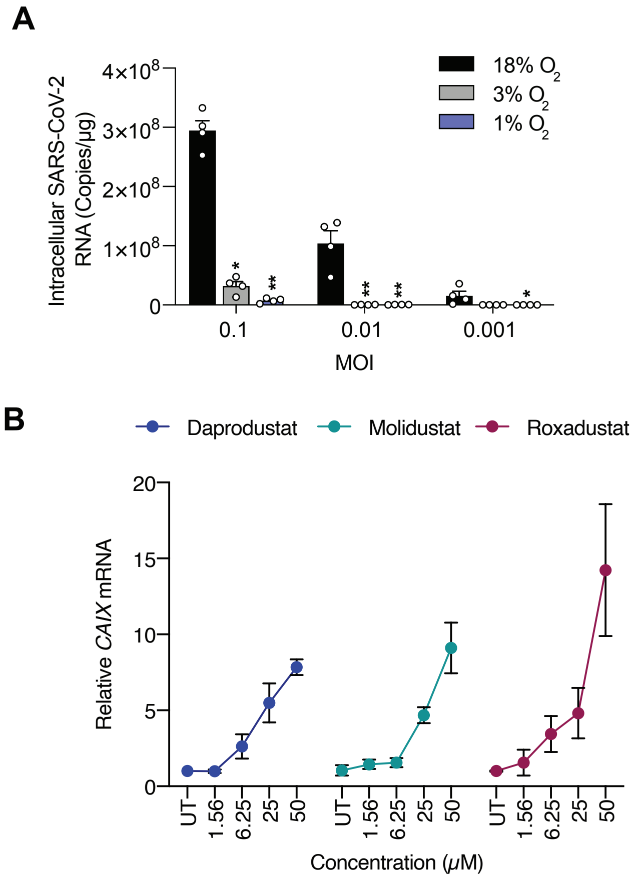

Fig.S4: Dose dependent Inhibition of SARS-CoV-2 by hypoxia and HIF prolyl hydroxylase inhibitors. [Related to Fig.2]. (A) Calu-3 cells were incubated at 18%, 3% or 1% O_2 for 24h prior to infection with SARS-CoV-2 at the indicated MOIs. Viral RNA was quantified from infected cells 24h post infection. (B) Calu-3 cells were treated increasing concentrations of Daprodustat (GSK1278863), Molidustat (Bay 85-3934) or Roxadustat (FG-4592), infected with SARS-CoV-2 and *CAIX* mRNA quantified by qPCR. All data is n=4 biological replicates and presented as mean ± S.D. with statistical significance determined by two-way ANOVA, * $p<0.05$ ** $p<0.01$.

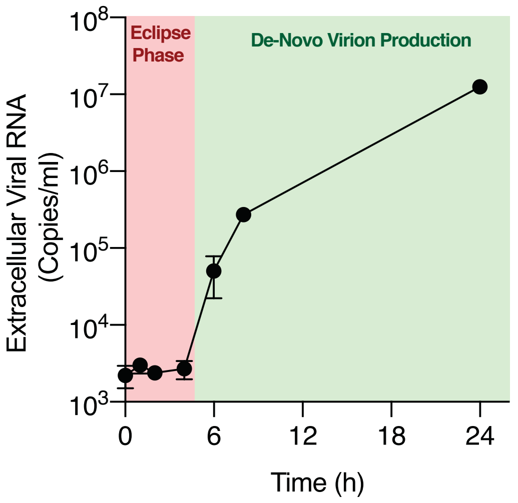

Fig.S5. Single step growth curve of SARS-CoV-2. [Related to Fig.4]. Calu-3 cells were inoculated with SARS-CoV-2 for 1h at an MOI of 1, unbound virus removed by washing and cells cultured in 18% O_2. At the indicated times extracellular samples were collected and SARS-CoV-2 RNA quantified by qPCR. Data is presented as mean ± range of n=2 biological replicates.

Supplementary Table 1: Probe sequences for quantification of SARS-CoV-2 RNA by smFISH [Related to STAR METHODS]

**Positive gRNA probes**
TAGATCGGCGCCGTAACTAT
TCCTTTATTACCGTTCTTAC
AGAAGAACCTTGCGGTAAGC
TACTGAATGCCTTCGAGTTC
AGCATCCGAACGTTTGATGA
TAGTAGTTGTCTGATTGTCC
GTCTTGTTGACCAACAGTTT
CTCATATTGAGTTGATGGCT
AGTAGTATGTAGCCATACTC
TCTAAATCAATGCCCAGTGG
GTAATTCAGATACTGGTTGC
CCTTTGAGTGTGAAGGTATT
GAGCAACATAAGCCCGTTAA
AGGTTGTTCTAATGGTTGTA
CATAGGGCTGTTCAAGTTGA
GCTTTTAGAGGCATGAGTAG
TGCGTGACAAATGTTTCACC
AAGGCTTTAAGTTTAGCTCC
CCCAACCGTCTCTAAGAAAC
AAGCCAATCAAGGACGGGTT
TTAGTTAGCCACTGCGAAGT
ACTGAACAACACCACCTGTA
GTAGGCCATTACAACTAGAT
AGTAGCCAAATCAGATGTGA
TTATAGCGGCCTTCTGTAAA
TTGACGTGCCTCTGATAAGA
TGCGGGAGAAAATTGATCGT
GGCGATCTCTTCATTAAGTT
GGTTGTCATTAAGACCTTCG
ACAACCTATGTTAGCGCTAG
ATAGGCACACTTGTTATGGC
TCCAAAGGCAATAGTGCGAC
AAGACTATGCTCAGGTCCTA
AGTAACCACAAGTAGTGGCA
TCACACTTCATGAGAGTTGA
GCACATTTGGTTGCATTCAT
CAAAGCCACGTACGAGCACG
GGTGACGCAACTGGATAGAC
CCTTGGTTGAATAGTCTTGA
TTTCAGAACGTTCCGTGTAC
CCAGTTGTTCGGACAAAGTG
TTACCAGCACGTGCTAGAAG
AATGCACTCAAGAGGGTAGC
GTTATCGACATAGCGAGTGT
TAAGCTCACGCATGAGTTCA
CTTCATAAGGATCAGTGCCA
CTCGTCGCCTAAGTCAAATG
GCGAACCTGTAAAACAGGCA
